# Plant water potential improves prediction of empirical stomatal models

**DOI:** 10.1371/journal.pone.0185481

**Published:** 2017-10-12

**Authors:** William R. L. Anderegg, Adam Wolf, Adriana Arango-Velez, Brendan Choat, Daniel J. Chmura, Steven Jansen, Thomas Kolb, Shan Li, Frederick Meinzer, Pilar Pita, Víctor Resco de Dios, John S. Sperry, Brett T. Wolfe, Stephen Pacala

**Affiliations:** 1 Department of Biology, University of Utah, Salt Lake City, Utah, United States of America; 2 Arable Labs, Princeton, New Jersey, United States of America; 3 Connecticut Agricultural Experiment Station, New Haven, Connecticut, United States of America; 4 Hawkesbury Institute for the Environment, Western Sydney University, Penrith, New South Wales, Australia; 5 Institute of Dendrology, Polish Academy of Sciences, Kórnik, Poland; 6 Institute of Systematic Botany and Ecology, Ulm University, Ulm, Germany; 7 School of Forestry, Northern Arizona University, Flagstaff, Arizona, United States of America; 8 Pacific Northwest Research Station, United States Forest Service, Portland, Oregon, United States of America; 9 Technical University of Madrid, Madrid, Spain; 10 Department of Crop and Forest Sciences and Agrotecnio Center, Universitat de Lleida, Lleida, Spain; 11 Smithsonian Tropical Research Institute, Balboa, Panama; 12 Department of Ecology and Evolutionary Biology, Princeton University, Princeton, New Jersey, United States of America; Estacion Experimental del Zaidin, SPAIN

## Abstract

Climate change is expected to lead to increases in drought frequency and severity, with deleterious effects on many ecosystems. Stomatal responses to changing environmental conditions form the backbone of all ecosystem models, but are based on empirical relationships and are not well-tested during drought conditions. Here, we use a dataset of 34 woody plant species spanning global forest biomes to examine the effect of leaf water potential on stomatal conductance and test the predictive accuracy of three major stomatal models and a recently proposed model. We find that current leaf-level empirical models have consistent biases of over-prediction of stomatal conductance during dry conditions, particularly at low soil water potentials. Furthermore, the recently proposed stomatal conductance model yields increases in predictive capability compared to current models, and with particular improvement during drought conditions. Our results reveal that including stomatal sensitivity to declining water potential and consequent impairment of plant water transport will improve predictions during drought conditions and show that many biomes contain a diversity of plant stomatal strategies that range from risky to conservative stomatal regulation during water stress. Such improvements in stomatal simulation are greatly needed to help unravel and predict the response of ecosystems to future climate extremes.

## Introduction

Anthropogenic climate change is expected to lead to more frequent and more severe droughts in many regions across the globe [1,2]. Both temperature-driven increases in evaporative demand and decreases in soil moisture are projected, which are known to stress plants substantially [3,4]. Severe drought plays a critical role in terrestrial carbon cycling, driving declines in plant productivity and potentially loss of carbon stocks if accompanied by widespread tree mortality, which has been observed on all vegetated continents in the past few decades [5–8] despite relatively modest changes in drought to date [9]. The response of the biosphere to climate extremes is one of the dominant uncertainties in the large spread of models around the fate of the terrestrial carbon sink in the 21^st^ century [7,10].

Yet mechanisms of plant drought response in global vegetation models are relatively simple and are in large part determined by leaf-level responses of stomata to environmental drivers [11,12]. Despite much progress in the past several decades [13–20], full mechanistic understanding of stomatal function remains incomplete, and thus many ecosystem models use empirical stomatal algorithms derived from leaf-level gas exchange [21–23], often during wet soil conditions. A growing number of ecosystem models include supply-side water limitation through simulating plant hydraulic transport and water potential [24–29], and thus it is likely to be instructive to test models that simulate leaf water potential against the standard empirical models [28]. Considering common empirical models, the Ball-Berry-Leuning is perhaps the most widely used empirical stomatal conductance model and gives stomatal conductance as a simple function of photosynthetic rate, atmospheric carbon dioxide concentration, and either relative humidity or vapor pressure deficit (VPD) [21,23]. Tuzet et al. propose a key modification of the Ball-Berry-Leuning model where the VPD dependence of stomatal conductance is replaced by a sigmoidal response to plant water potential [30]. Medlyn et al. derive a model similar to the Ball-Berry-Leuning model by optimizing carbon gain for a given water lost per the classic Cowan-Farquhar theory [31], leading to a model that is more interpretable but with largely the same functional dependencies on photosynthesis, CO_2_ concentrations, and VPD [32,33]. Finally, a recent study proposed a new empirical model based off an alternate optimization strategy for stomata—that stomata maximize carbon gain minus the costs of hydraulic damage—that is consistent with plant competition for water and includes both a VPD and leaf water potential sensitivity [34].

These empirical stomatal conductance models tend to perform remarkably well in lab experiments and also reasonably well during short-term periods of water stress, but have rarely been tested during severe or long-term (e.g. weeks to years) drought [3,35]. Also, models based on the theoretical predictions from the Cowan & Farquhar formulation [31] of optimal stomatal behavior are challenging to implement in ecosystem models. This is because the marginal water cost of carbon (often termed λ) is observed to vary widely and non-linearly within a single species in response to soil water stress [36–38]. One piece of evidence that the classical empirical models may not be adequate during drought is that, with recent exceptions of models with water transport [26,29], most large-scale ecosystem models include a rarely-tested soil moisture stress function, that shuts stomata as soil water potential falls [39–41].

Decades of research have detailed that stomata respond to leaf water potential directly [13,42–44]. Temporal variation in leaf water potential is influenced both by VPD and soil water potential, and the functional form of stomatal response to leaf water potential differs distinctly between these two drivers and across species [28]. Indeed, the stomatal response to leaf water potential is likely one of the central elements of a plant’s drought response strategy [44–49]. The water potential sensitivity of stomata is predicted to be tightly coupled to species’ xylem anatomy and the xylem’s vulnerability to cavitation [28,37,48,50]. Xylem traits will thus influence a plant’s stomatal strategy and the coupled hydraulic-stomatal continuum is likely to be critical in predicting which plants might be vulnerable to drought-induced tree mortality [11,51–53]. It is increasingly apparent that some representation of plant hydraulics and its effects on stomata will be needed to capture the manifold and species-specific impacts of climate change-driven droughts [26,29,35,48,53].

In this paper, we compile datasets of stomatal conductance in response to varying environmental conditions and leaf water potential from 34 woody plant species around the world to ask: 1) What is the marginal dependence of stomatal conductance on leaf water potential, after factoring out the VPD and photosynthetic changes, which would help constrain the soil moisture function in ecosystem models? 2) How well do existing stomatal conductance models perform during drought and are there systematic biases at high leaf temperatures, high vapor pressure deficits or low soil water potentials? 3) Does a stomatal model that builds on current empirical models but includes the marginal effect of leaf water potential improve prediction of stomatal conductance, both overall and also during dry conditions?

## Methods

### Gas exchange and water potential datasets

We performed an extensive literature search to identify studies that contained both measurements of stomatal conductance (*g*_s_) and its driving variables and leaf water potential. To be considered, a study needed to include: 1) direct measurements of *g*_s_ through gas exchange (e.g. Li-COR 6400) or porometry methods, 2) measurement of atmospheric CO_2_ concentrations concurrently, 3) measurement of VPD (using either air temperature or leaf temperature), and 4) concurrent measurements of twig or leaf water potential. We drew upon a recent synthesis of *g*_s_ datasets from around the world [33] for those containing leaf water potential measurements. We next contacted researchers who had published in this area to ask for recommendations of relevant studies and datasets. Finally, we performed a suite of literature searches through Google Scholar and ISI Web of Science using various permutations of “stomatal conductance,” “gas exchange,” “water relations,” “water potential,” “drought,” and “stomatal behavior.” We included only studies by authors who generously agreed to share their raw measurements because this allowed a much greater sample size per species (critical for partial dependency and model selection analyses—see below) than the use of published means.

In the end, we obtained datasets on 34 species from around the world (Table 1; Table A in S1 File) from 15 studies. All species were C3 plants, including 6 gymnosperms and 28 angiosperms from tropical, temperate, and boreal regions. Datasets from 9 species came from studies of potted trees, while 25 were from trees grown and measured in field conditions. All species except 3 recorded either midday water potential or water potential concurrent with stomatal conductance measurements. The remaining 3 species had only measurements of pre-dawn water potential, which was used in place of midday water potential (*Quercus ilex*, *Populus balsamifora*, *Pistacia lentiscus*) to account for soil moisture’s effects on stomatal conductance. Twenty-four species had concurrent measurements of assimilation. Almost all datasets (N = 32 species) covered dry conditions where substantial water stress occurred, as evidenced by water potential measurements that would cause greater than 10% embolism based on published xylem cavitation vulnerability curves for these species (Weibull-functions consistent with the water potentials at which 50% and 88% of stem hydraulic conductivity is lost). Xylem trait values were drawn either from the study itself (N = 8 spp) or the Xylem Functional Traits dataset [54]. We used this xylem vulnerability curve to calculate the percent loss in stem hydraulic conductivity at the most negative leaf water potential observed in the dataset. While transpiration-induced disequilibrium between leaf and stem water potential could potentially lead to artificially high estimates of losses in conductivity, we believe that this is likely minimal because transpiration rates should have been minimal at the most negative leaf water potentials.

**Table 1:** Species included in the study, their Akaike information criterion (AIC) values and R^2^ values during model selection. Species with no values were not included in the model selection analyses because photosynthesis was not measured directly (but were included in the RandomForest analyses). In AIC values, bolded values indicate that a given model for a species was the most likely (delta-AIC > 3) for that species. Models include the Medlyn (M), Ball-Berry-Leuning (BBL), Ball-Berry-Leuning with hydraulic addition (BBL.H) and Tuzet (T) models (see Methods).

Species	AIC M	AIC BBL	AIC BBL.H	AIC T	R^2^ M	R^2^ BBL	R^2^ BBL.H	R^2^ T
*Acer campestre*	-154.82	-156.95	-159.49	-161.50	0.82	0.84	0.86	0.86
*Acer pseudoplatanus*	-141.70	-146.76	-143.21	-135.57	0.75	0.79	0.79	0.73
*Alphitonia excelsa*	-271.80	-281.04	**-309.06**	-294.86	0.55	0.58	0.65	0.62
*Anacardium excelsum*								
*Annona hayesii*	-102.38	-105.33	-108.61	-110.81	0.29	0.36	0.46	0.46
*Astronium graveolens*								
*Austromyrtus bidwillii*	-154.10	-153.27	-151.46	-147.90	0.44	0.46	0.49	0.41
*Brachychiton australis*	-193.88	-193.56	-189.56	-184.38	0.40	0.41	0.41	0.37
*Bursera simaruba*								
*Carpinus betulus*	-186.73	-188.61	-184.61	-186.60	0.73	0.75	0.75	0.75
*Cavanillesia platanifolia*								
*Cochlospermum gillivraei*	-198.96	-206.95	-202.95	-208.78	0.52	0.58	0.58	0.60
*Cojoba rufescens*								
*Cordia alliodora*								
*Corylus avellana*	-143.78	-142.27	-151.20	-151.20	0.85	0.85	0.90	0.89
*Eucalyptus globulus*	-97.66	-108.44	-115.23	-61.15	0.80	0.83	0.86	0.69
*Ficus insipida*								
*Fraxinus excelsior*	-158.03	-156.81	-154.81	-147.64	0.85	0.85	0.86	0.82
*Genipa americana*								
*Juniperus monosperma*	-3171.58	-3429.01	**-3554.22**	-3516.62	0.77	0.85	0.88	0.87
*Juniperus osteosperma*	-203.74	-200.21	-208.29	**-211.93**	0.73	0.72	0.80	0.81
*Phillyrea angustifolia*	-66.91	-72.63	**-75.82**	-69.79	0.67	0.79	0.86	0.78
*Picea abies*	-1311.60	-1308.62	-1304.60	-1293.58	0.41	0.40	0.40	0.39
*Pinus edulis*	-3159.62	-3235.26	**-3259.00**	-3196.97	0.79	0.82	0.83	0.81
*Pinus ponderosa*	-627.43	-627.59	-624.32	-601.72	0.74	0.74	0.74	0.69
*Pistacia lentiscus*	-107.76	-106.70	-102.70	-102.72	0.72	0.73	0.73	0.71
*Populus balsamifora*	-52.23	-50.38	-52.31	-54.30	0.49	0.49	0.59	0.59
*Populus tremuloides*	-162.46	-172.74	-171.55	-143.60	0.42	0.56	0.59	0.18
*Prosopis velutina*	**-65.55**	-62.42	-59.45	-60.28	0.42	0.40	0.42	0.40
*Quercus douglasii*	-393.15	-440.33	-437.75	-405.41	0.67	0.76	0.76	0.70
*Quercus gambelii*	-66.83	-67.64	-63.64	**-73.89**	0.90	0.92	0.92	0.96
*Quercus ilex*	-648.62	-646.21	-642.21	-648.10	0.91	0.91	0.91	0.91
*Schefflera morototoni*								
*Tapirira guianensis*								

### Partial dependence analyses

To quantify the partial dependence of stomatal conductance on leaf water potential—i.e. the marginal effect of leaf water potential when other potential co-drivers, such as photosynthesis, VPD, and atmospheric CO_2_ concentrations are held constant—we used the RandomForest machine learning algorithm [55]. In essence, this marginal curve reflects the partial dependency of g_s_ on soil water potential, all else constant. This algorithm uses bootstrapped subsamples of the full dataset to perform regression tree analysis on a large number (N = 500) of trees, which are then aggregated. We chose this algorithm because 1) it performs well among machine-learning algorithms, 2) it makes no assumptions around the distribution (e.g. normality) of the input data, 3) it makes no assumptions on the functional form of the relationship between independent and dependent variables (e.g. linear, non-linear, etc), and 4) it can handle interactions between independent variables [55,56].

For each species, a RandomForest model was fit using 500 regression trees and a minimum node size of 5 (i.e. nodes cannot be split if they have fewer than 5 observations). Each model simulated stomatal conductance as a function of leaf water potential, vapor pressure deficit, assimilation, and atmospheric CO_2_ concentrations. We excluded species that had fewer than three measurements of leaf water potential (N = 5 species), as this would not allow accurate reconstruction of the partial dependence on leaf water potential. For the 10 species without assimilation measurements, we modeled assimilation using the Farquhar et al. [57] photosynthesis model for the RandomForest analysis only using the “plantecophys” package in the R statistical software. The RandomForest algorithm yields an estimate of out-of-bag prediction error, which quantifies model performance on subsets of the data on which the model was not trained. Next, we isolated the marginal effect of leaf water potential while holding other independent variables constant via a partial dependency analysis on the RandomForest model. To standardize comparison of the marginal effect of leaf water potential curves on species with different stomatal conductance ranges and different degrees of water stress, we re-scaled each marginal curve to the degree of stomatal closure that occurred for each species during water-stressed conditions (i.e. scaling between highest measured gs and lowest measured gs when light and atmospheric CO_2_ were high). Thus, if a species was observed to have maximum gs values of 100 mmol*m^-2^*s^-1^ and minimum gs values during water-stressed conditions of 40 mmol*m^-2^*s^-1^, that species’ partial dependency curve was scaled between 0.4 and 1.

### Comparison of stomatal algorithms

On the 24 species subset that had direct measurements of assimilation, we compared the predictive accuracy of four stomatal algorithms during all conditions and during dry conditions with low soil water potentials. The algorithms were: 1) the optimal-empirical model described in Medlyn et al. [32]:

$$g_{s}=1.6*\frac{A}{C_{s}}*\left(1+\;\frac{g_{1}}{\sqrt{D}}\right)$$ (1)

where A is photosynthesis, C_s_ is CO_2_ concentration at the leaf surface, D is VPD, and g_1_ is a species-specific parameter; 2) the standard Ball-Berry-Leuning model, but excluding the g_0_ term, as in [32,33]:

$$g_{s}=\;\frac{\propto A}{\left(C_{s}-\Gamma^{*}\right)\left(1+\frac{D}{d_{1}}\right)}$$ (2)

where Γ* is the CO_2_ compensation point and α and d_1_ are species-specific parameters; 3) the Tuzet et al. update of the Ball-Berry-Leuning where the VPD dependence is replaced by a leaf water potential sigmoidal curve, fit here as a Weibull function, due to its flexibility and widespread observations of Weibull forms in xylem conductivity functions:

$$g_{s}=\;\frac{\propto A*e^{-{\left(\frac{\psi }{c}\right)}^{b}}}{\left(C_{s}-\Gamma^{*}\right)}$$ (3)

where c and b are species-specific parameters and *ψ* is leaf water potential. And 4) the initial Ball-Berry-Leuning model combined with a Weibull sensitivity to leaf water potential, which is a recent variant proposed by [34] which derives the model similar to Medlyn et al. [32] but from a carbon maximization optimization, rather than the classical marginal water use efficiency optimization:

$$g_{s}=\;\frac{\propto A*e^{-{\left(\frac{\psi }{c}\right)}^{b}}}{\left(C_{s}-\Gamma^{*}\right)\left(1+\frac{D}{d_{1}}\right)}$$ (4)

We note that Eq 4 is a combined model of Eqs 2 and 3. We emphasize that the first two models account for only atmospheric effects (VPD and leaf surface CO_2_ concentration) on stomatal conductance while the third and fourth models begin to include soil moisture stress through leaf water potential. We used a Nelder-Mead optimization algorithm to find the best parameters for each model that minimized the sum of squared errors between the predicted and observed stomatal conductance. Because this method is most effective at finding local minima, we initiated each species from multiple initial conditions to ensure that the algorithm had found the global minima. This algorithm finds the best parameters that fit the entire dataset of stomatal conductance for each species and model combination.

Because the stomatal models have different numbers of parameters, we performed model selection with the R^2^ and the root-mean-squared error (RMSE) of the model and the Akaike information criterion (AIC). AIC allows inference on the relative quality of statistical models by estimating the information lost by different models, while accounting for different numbers of parameters between models. The combination of these three metrics allows quantification of the relative improvement in predictive power (e.g. % change in R^2^ or RMSE) and standardized model selection among models with different numbers of parameters. AIC differences between models provide inference in the relative likelihood of a given model, with differences of >3 generally considered to be evidence for choosing one model over another [58]. We tested for any potential biases of collinearity between VPD and leaf water potential through two methods: 1) comparing the AIC improvement in models with both variables (Eq 4) to the correlation between the two variables and 2) using variance inflation factors from multiple linear regressions involving all variables.

To quantify potential biases during dry conditions, we examined the residuals of the predicted versus observed stomatal conductance of each model for each species. We performed linear regression on the residuals compared to leaf temperature, VPD, and predawn plant water potential as a proxy for soil water potential. A statistically significant slope indicates that there is indeed a persistent bias in prediction of stomatal conductance at one or both ends of the predictor variable. We quantified the degree of bias by looking both at the slope of these regressions and, more critically for drought conditions, calculating the % over or under prediction of each model on each species during dry soil conditions, defined as the 10th percentile of soil water potentials.

## Results

### Stomatal dependence on leaf water potential

**Fig 1:**
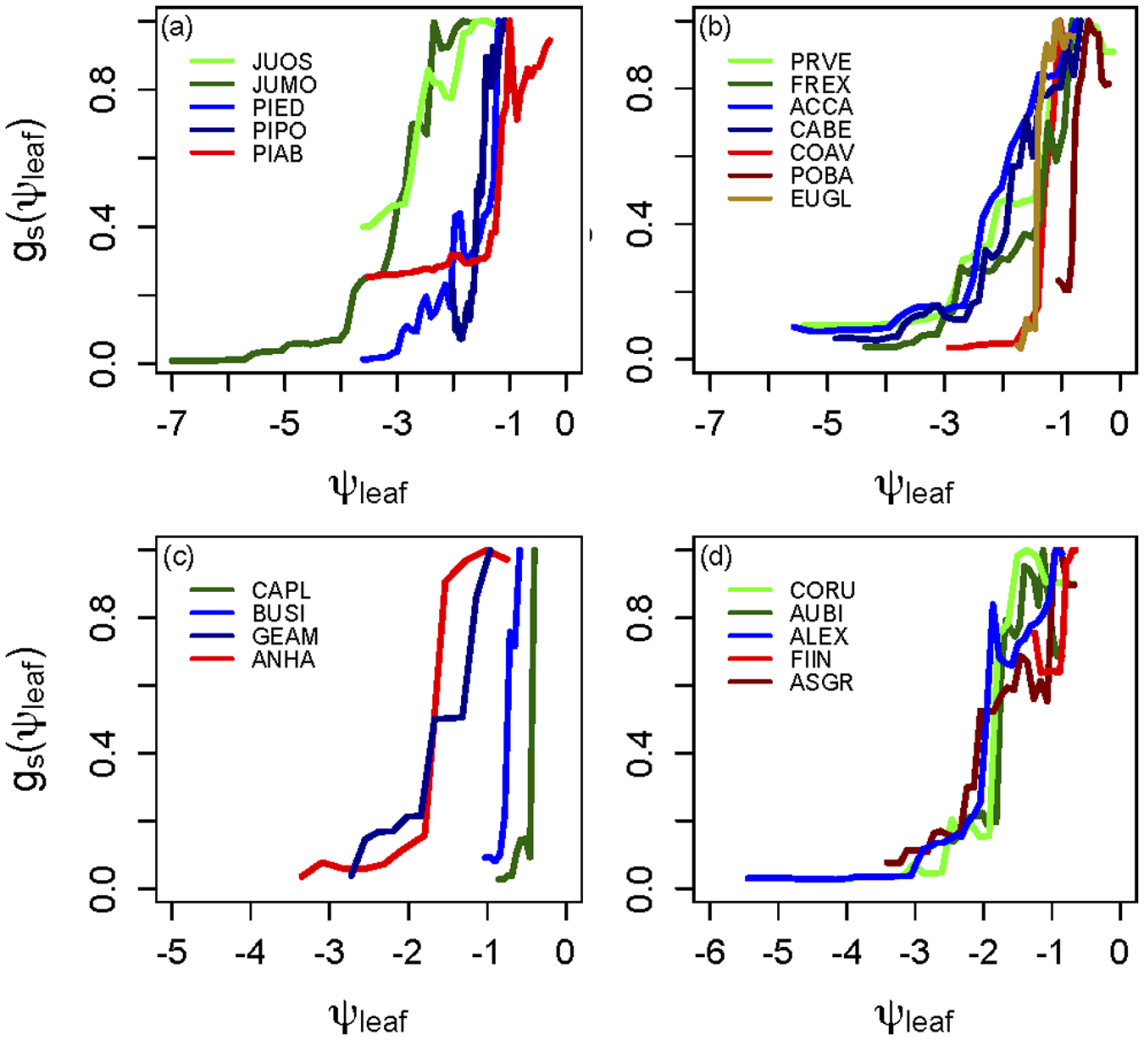
Partial dependency of stomatal conductance (g_s_) on measured leaf water potential (ψ_leaf_) in (a) temperate gymnosperm species, (b) temperate angiosperm species, (c) tropical deciduous species, and (d) tropical evergreen species. Y-axis values are scaled from 0–1 by the percentage of stomatal closure reached. Species codes are the first two letters of the genus and the first two letters of the species from Table A in S1 File.

**Fig 2:**
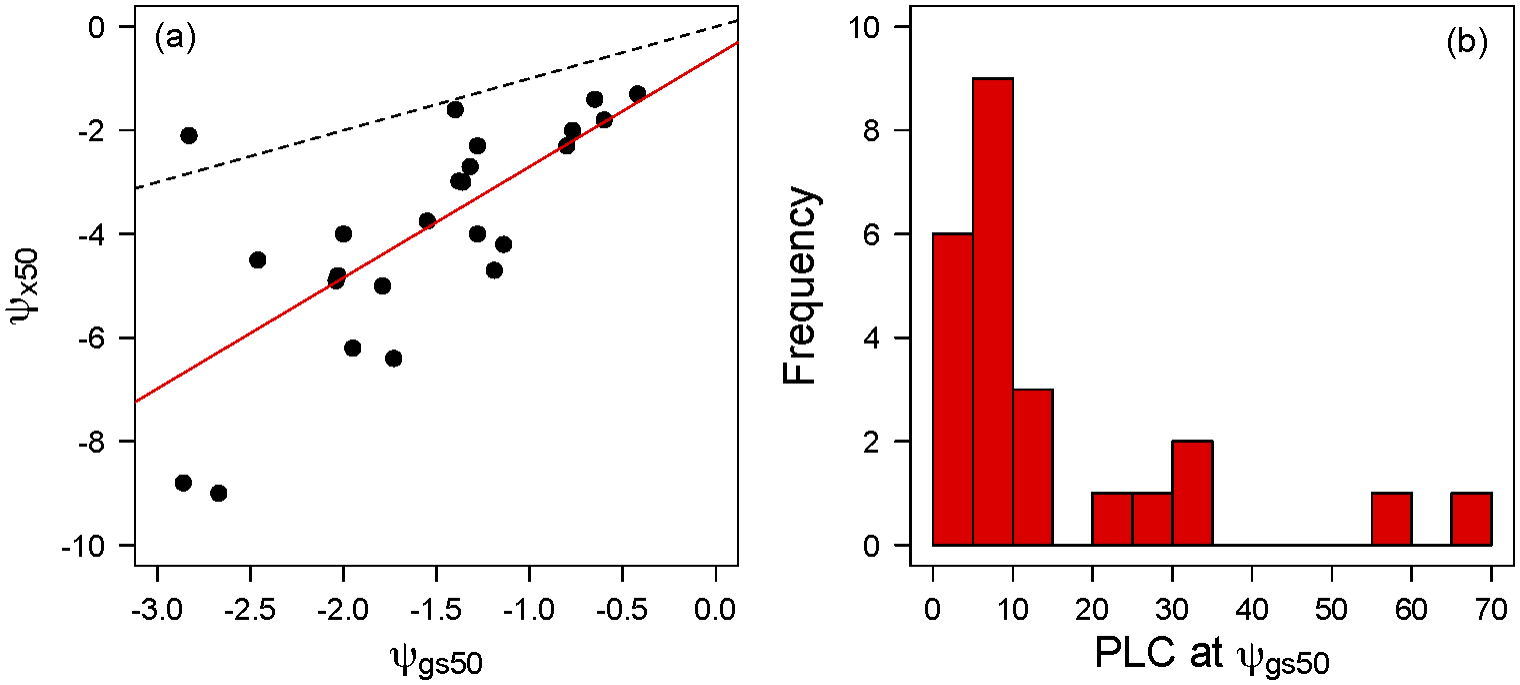
(a) Relationship across species between the water potential at which 50% of stomatal conductance is lost (ψ_gs50_) and the water potential at which 50% of stem hydraulic conductance (ψ_x50_) is lost. Black line is the 1:1 line and red the OLR regression best fit. (b) Histogram of the percent loss of stem hydraulic conductance (PLC) at the water potential at which 50% of stomatal conductance is lost (ψ_gs50_).

The RandomForest algorithm produced models with strong predictive power (mean R^2^ = 0.62), approaching that of the standard stomatal conductance models (mean R^2^ = 0.65–71), and also partial dependencies against assimilation, CO_2_ and VPD that are directly consistent with those represented in the standard empirical stomatal conductance models (see Fig A-C in S1 File for representative species). This provides high confidence that the machine learning algorithm generated robust, meaningful, and interpretable results and similarly implies empirical confidence in the structural form of the equations in the models. The marginal dependence of stomatal conductance on leaf water potential, when holding VPD constant (i.e. stomatal sensitivity to soil water potential-driven changes in leaf water potential), most closely resembled a Weibull curve for most species (Fig 1). This partial dependence curve was interpretable and consistent with the expected relationship (either Weibull, sigmoidal, or negative-exponential) for 26 of 29 species. For the remaining 3 species, the curves fluctuated erratically (Fig D in S1 File) and these species were not included in comparisons of species such as the one in Fig 2.

Species displayed systematic variance in their soil moisture stress-driven g_s_(ψ_leaf_) curves that are consistent with their documented drought response strategies. For example, in temperate gymnosperm species, the two pine species (*Pinus edulis* and *Pinus ponderosa*) considered to be largely isohydric showed rapid reduction in g_s_ with falling leaf water potentials, while the two juniper species (*Juniperus monosperma* and *Juniperus osteosperma*) observed to be largely anisohydric [59] showed much delayed and less sensitive g_s_ regulation with declining water potentials (Fig 1a). In addition, physiologically similar species showed similar g_s_(ψ_leaf_) responses even with only partial water stress observed in some cases. For example, *Juniperus osteosperma* only experienced modest water stress with ~50% declines in g_s_ but its g_s_(ψ_leaf_) largely followed that of the similar *Juniperus monosperma* that experienced much more severe water stress.

Species showed a strong relationship between stomatal regulation in response to water potential and xylem characteristics. There was a significant relationship between the water potential at 50% stomatal conductance loss driven by soil moisture stress and that at 50% stem xylem hydraulic conductance loss (R^2^ = 0.49, t = 4.6, p<0.0001) (Fig 2a). Because these two curves have a similar functional form—Weibull curves for most species—comparison of their midpoints also gives a sense of the relative offset between them. Both the slope of the relationship and that all species except one fell below the 1:1 line indicates that in most species stomatal conductance is down-regulated well-before substantial (e.g. 50%) embolism occurs in the stem xylem [60] (Fig 2a). Thus, the percent loss of stem hydraulic conductivity that occurs at the ψ_gs50_ point is under 15% for most species (mean: 14.9%; median: 9.1%) (Fig 2b), with the most prominent exception being *Quercus douglasii*. *Quercus douglasii* was the only species in our sample with an R-shaped xylem vulnerability curve [61], and this should be interpreted with caution because the data came from a study on seedlings whereas the gas exchange and water potential data used here was measured on mature trees.

### Comparison of stomatal algorithms

**Fig 3:**
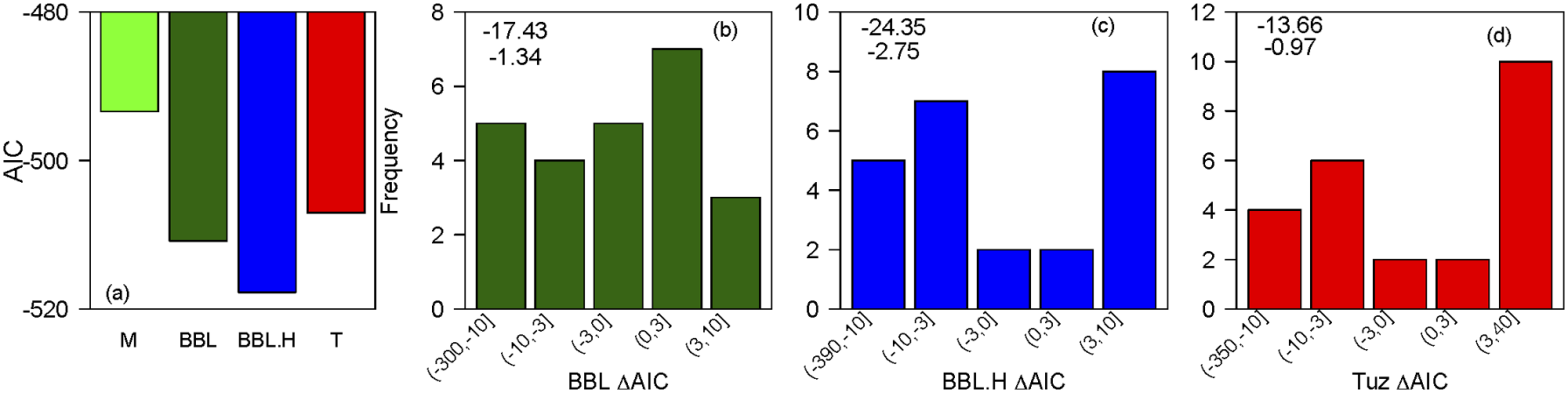
(a) Akaike Information Criterion (AIC) value average across all 24 species for the Medlyn (M), Ball-Berry-Leuning (BBL), Ball-Berry-Leuning plus Hydraulics (BBL.H), and Tuzet (T) stomatal conductance models. (b) Frequency of species’ delta-AIC values between the BBL and M models. (c) Frequency of species’ delta-AIC values between the BBLH and M models. (d) Frequency of species’ delta-AIC values between the T and M models. In each of (b-d), the top number is the mean delta-AIC across all species and the lower number is the median delta-AIC.

Model selection analysis revealed that the Ball-Berry-Leuning model with the hydraulic addition (BBL.H) was selected as the most likely model for most species with a large AIC signal (Fig 3a; Table 1). The Ball-Berry-Leuning model with the hydraulic addition had a mean AIC difference of -24.3 from the Medlyn model, -10.3 from the Tuzet model, and -7.9 from the standard Ball-Berry-Leuning model, giving its relative likelihood of being the “best” model (that minimizes information loss) of >0.999, >0.997, and 0.984 respectively (Fig 3a). These mean differences, however, were partially influenced by a few species with very large improvements in the BBL.H, and thus the median differences were less stark but still showed the same pattern (Fig 3b–3d). Considering species separately, AIC differences revealed significant differences among the models in 33% of the species (8 of 24) (Table 1). Assuming the standard rule-of-thumb that AIC differences of >3 are considered grounds for preference of one model over another [58], the BBL.H model was the most likely model for 62.5% (5/8) of the species where one model was unequivocally the most likely, with the Tuzet model most likely for 25% (2/8) and the Medlyn model for 12.5% (1/8) (Table 1). Collinearity between VPD and leaf water potential is unlikely to have driven the increased predictive ability of the BBL.H model because we observed no correlation between the AIC differences and VPD-ψ_leaf_ correlation across species (p = 0.71) and all species’ variance inflation factors were <3, which indicates little risk of collinearity in predictor variables (VIF>10 is generally considered large collinearity requiring correction) [62]. Further, we saw no evidence of correlations among model parameters (p>0.2 in all cases).

**Fig 4:**
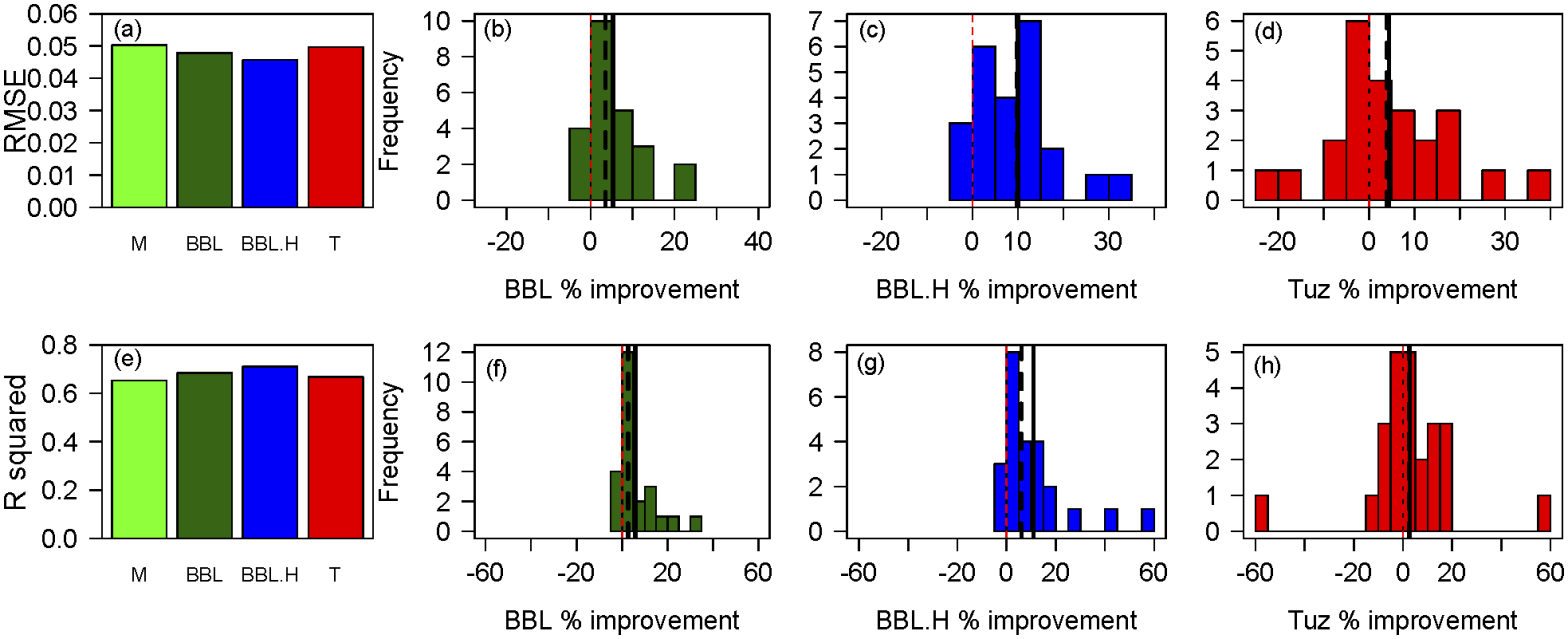
(a) Root mean squared error (RMSE) and (e) OLS regression R^2^ between predicted and observed stomatal conductance averaged across all 24 species for the Medlyn (M), Ball-Berry-Leuning (BBL), Ball-Berry-Leuning plus Hydraulics (BBL.H), and Tuzet (T) stomatal conductance models. (b and f) Frequency of species’ % improvement in RMSE and R^2^ respectively between the BBL and M models. (c and g) Frequency of species’ % improvement in RMSE and R^2^ respectively between the BBLH and M models. (d and h) Frequency of species’ % improvement in RMSE and R^2^ respectively between the T and M models.

Predictive differences between the four models revealed the same pattern that, while all models performed relatively well, the BBL.H model had consistently lower RMSE values and higher R^2^ values (Fig 4a and 4e). Compared to the model with the lowest predictive ability, the BBL.H model showed a 6–11% improvement in R^2^ and RMSE (Fig 4c and 4g) and a ~5% improvement over the second best model, the standard BBL model (Table 1). Species varied substantially in their improvements in predictive power, with several species having a >30% increase in predictive power in the BBL.H model. The AIC analysis reveals that the improvements in predictive power were usually statistically significant and thus not just a consequence of an additional parameter. Considering all three metrics, we observe strong evidence that the BBL.H model performs significantly better in the majority of species, with the standard BBL model and the Tuzet model performing roughly equally well as second-best models.

### Performance during drought conditions

**Fig 5:**
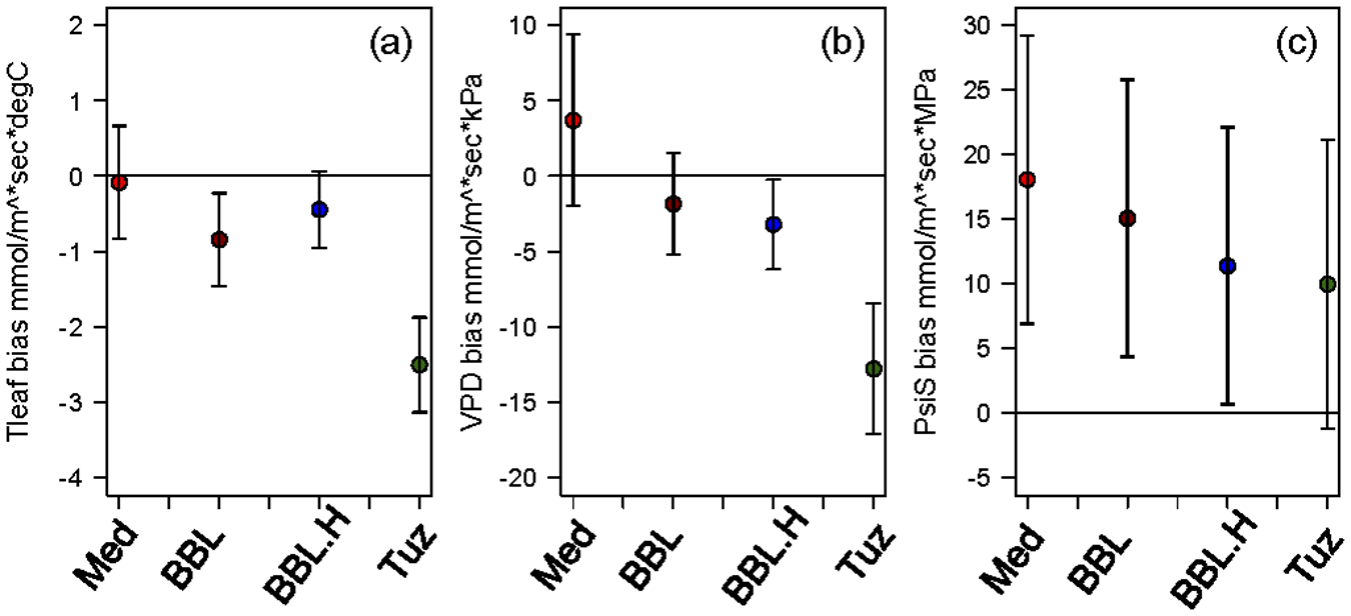
(a) Slope between the residuals of observed versus predicted stomatal conductance and leaf temperature (Tleaf), (b) vapor pressure deficit (VPD), and (c) soil water potential (PsiS). Negative values in the Tleaf and VPD plots indicate over-prediction of g_s_ during dry conditions (high Tleaf and high VPD), whereas positive values in the PsiS plot indicates over-prediction of g_s_ during dry conditions. Models are Medlyn (red), Ball-Berry-Leuning (darkred), Ball-Berry-Leuning plus the hydraulic term (blue) and Tuzet (green). Error bars are +/- 1 S.E.

The residuals of the predicted-versus-observed g_s_ regressions show that many of the models are biased because they overpredict stomatal conductance during conditions of low soil water potential, high VPD, and high T_leaf_ (Fig 5). For the response of stomatal aperture to VPD and T_leaf_, the Tuzet model had the largest bias, while the Medlyn, BBL, and BBL.H had relatively low biases. For soil water potential, the largest biases were observed in the Medlyn model, with the BBL and BBL.H models as intermediate, and the lowest biases in the Tuzet model. We note that because all models’ parameters were optimized using all data for each species, that even the “best fit” model that includes dry conditions still tends to overpredict g_s_ in these conditions. This is likely because there are generally more g_s_ measurements during wet/benign periods in most datasets and minimizing the larger absolute errors at high g_s_ values would tend to be favored by parameter fitting algorithms. However, focusing prediction error at drier periods in the dataset could have an attendant trade-off of less accurate prediction during wetter periods.

**Fig 6:**
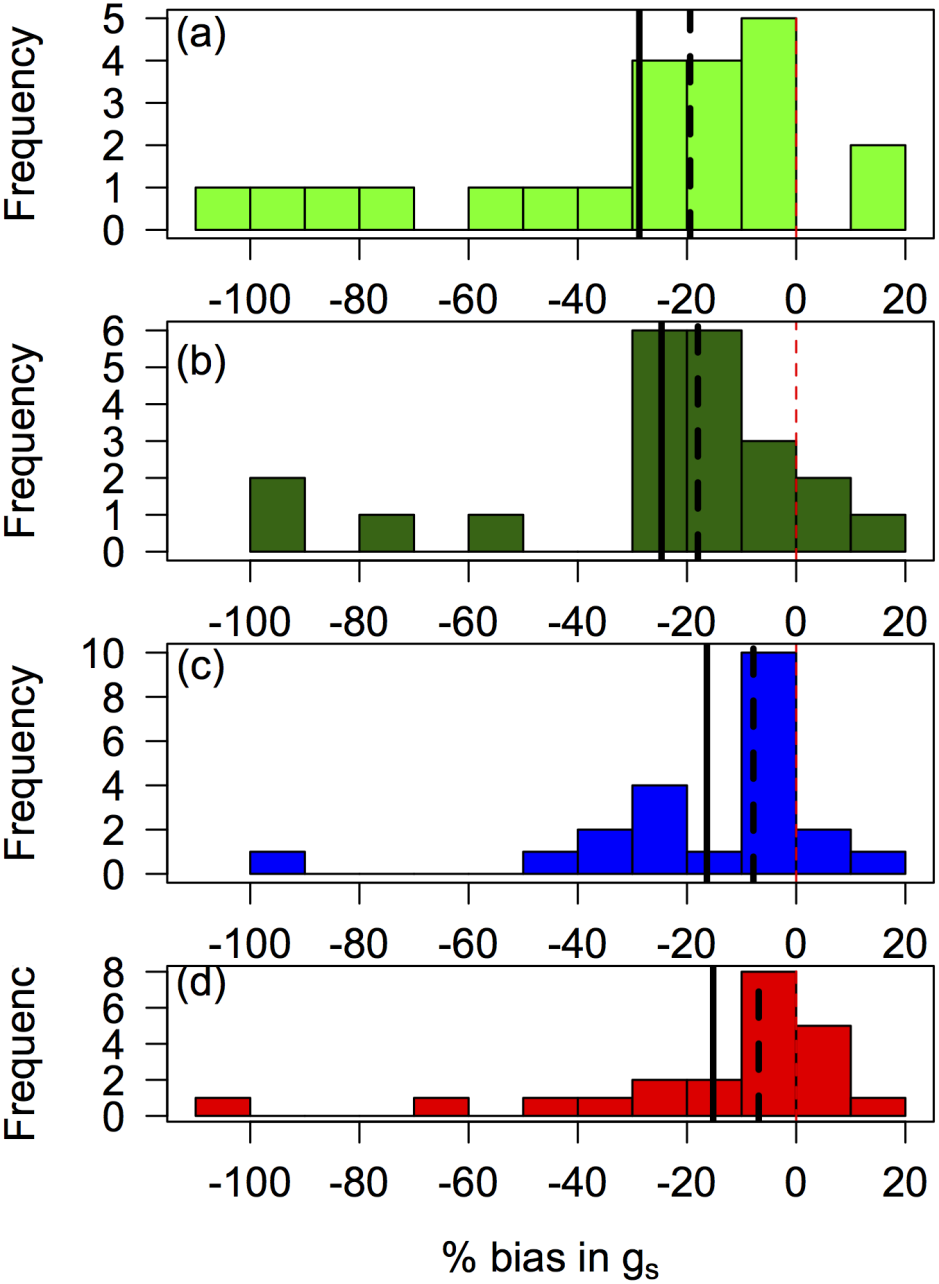
Histogram of species’ percent bias in prediction of stomatal conductance at the 10^th^ percentile of soil water potential. Negative numbers mean over-prediction of g_s_ by models compared to measured values. Solid line is the mean across species and dashed line the median. Models are (a) Medlyn model, (b) Ball-Berry-Leuning model, (c) Ball-Berry-Leuning plus Hydraulics model, and (d) Tuzet model.

For all models, the largest biases occurred during periods of low soil moisture (Fig 6). Considering the 10^th^ percentile of soil water potential, the Medlyn model overpredicted g_s_ by an average of 28.7% (median: 19.4%); the BBL model overpredicted by an average of 24.7% (median: 18%); and the BBL.H and Tuzet models overpredicted by an average of 16.4% and 15.2% (medians: 7.8% and 6.9%) respectively (Fig 6). Thus, the BBL.H and Tuzet model greatly reduced the biases during dry soils by 40–50%, although not removing the bias altogether, and the Tuzet model showed higher bias at high VPDs.

## Discussion

We show here that inclusion of leaf water potential in stomatal conductance models both improves their predictive power and substantially reduces the biases observed during dry conditions in the current standard empirical models. The observed prevalence of Weibull-shaped functions in the partial dependency of g_s_ on leaf water potential strongly agrees with the published curves using dry-down or dehydration experiments [44] and this curve arises from the fundamental water transport equations [34]. This partial dependency analysis provides empirical support for the functional form of soil moisture stress on stomatal conductance and can guide the formulation of these functions in ecosystem models. The shape of the g_s_(ψ_leaf_) driven by soil moisture stress likely arises distinctly from the xylem vulnerability curve shape and the marginal cost of opening stomata [34,63]. Our results further confirm that a Weibull-like (or sigmoidal) function is most appropriate for incorporation into empirical stomatal models, although future research will be needed to determine the degree to which g_s_(ψ_leaf_) curves vary within species across populations, environmental gradients, and seasons or years. Even relatively “fixed” hydraulic traits, such as the water potential at 50% loss of stem hydraulic conductance, show fairly substantial within-species variation, with larger variation in angiosperms than gymnosperms [64].

In addition, the diversity in g_s_(ψ_leaf_) curves of trees from different biomes (Fig 1) reveals that there is likely a wide diversity of stomatal strategies found in most biomes across the world. This diversity of g_s_(ψ_leaf_) curves is visible in the wettest tropical forest species measured here (e.g. Fig 1c) and in the driest woodland with only two species (*Pinus edulis-Juniperus monosperma*) (Fig 1a). This may have important implications for land-atmosphere interactions, particularly during droughts. Current vegetation models have only one set of stomatal conductance parameters per plant functional type (and parameters are often identical across plant functional types) [33], which implies a need for a greater range of hydraulically-defined plant functional types. If diversity in plant stomatal and hydraulic strategies is prevalent in many ecosystems, ecosystem response to water deficits could be fundamentally different than what would be predicted by ecosystem models.

Our estimation of the g_s_(ψ_leaf_) curves also shows a strong coupling between stomatal responses to water stress and xylem vulnerability to cavitation (Fig 2a). This coupling has been documented in other studies [48,60,65,66] and highlights the critical linkages between plant water transport and gas exchange responses to water stress. Comparison between stomatal and xylem curves also emphasizes that most plants curtail stomatal conductance at xylem water potentials well above the corresponding ψ_x50_. This is strongly consistent with much research indicating that plants regulate stomata partially to manage hydraulic damage [43,44,48,67] and avoid the fitness consequences incurred by low water potential, including decreased growth and increased mortality rates [53,68–70]. Furthermore, Fig 2a highlights two useful axes of drought response strategies. The first is the widely recognized shifts in xylem vulnerability (ψ_x50_; y axis of Fig 2a) across ecosystems with aridity [54,71,72]. The second highlights the gap between the g_s_(ψ_leaf_) curve and the xylem vulnerability curve (regression line and spread around it in Fig 2a), representing a risky-versus-conservative difference in strategy, where more rapid g_s_ closure as ψ_leaf_ falls would be a “conservative” strategy to avoid embolism.

We observed evidence that including leaf water potential in the standard empirical models of stomatal conductance increased predictive power in the majority of the 33% of species where detectable differences occurred between stomatal models. Perhaps more importantly, including leaf water potential decreased overprediction biases during dry conditions. Because the functional forms of the BBL, Tuzet, and BBL.H model are identical aside from their treatment of drought responses, we can draw the inference that including both VPD and leaf water potential was the most likely model across all species, with the BBL (VPD only) and Tuzet (water potential only) models having roughly similar, but less accurate, predictive ability. Why would including both VPD and water potential improve predictive power? This is likely because mixing of outside air (and its VPD) with the vapor pressure in the substomatal pore indicates that the effective water potential that stomata “sense” is likely some weighted average of the two that varies with stomatal aperture [14,18,73].

Our results reveal the importance of considering plant water transport and hydraulics in simulation of carbon and water fluxes in models and emphasize that empirical models that include leaf water potential like those tested here or even more detailed mechanistic models [14,16,18] have promising potential to improve predictions. The documented over-prediction of g_s_ at the leaf level during drought conditions may occur at ecosystem scales or may be currently crudely accounted for using the “soil moisture stress” function present in most ecosystem models, which to our knowledge has not been tested against data in most models. We posit that including leaf water potential in the empirical stomatal model (e.g. BBL.H presented here) would allow replacing of this previous equation, higher parsimony, and higher fidelity to the widely documented mechanism of leaf water potential’s effect on stomatal conductance. Indeed, dynamic simulation of water potential from climate and edaphic data and plant hydraulic traits is now possible [12,24–29] and reproduces stomatal response to water deficits with remarkable accuracy [28]. Furthermore, many of the key hydraulic traits—such as the xylem vulnerability curve—are known for a diversity of species across many biomes [64,72]. Ultimately, linking of plant hydraulics and stomatal conductance will enable improved prediction of plant responses to climate extremes in a rapidly changing climate.

## Supporting information

S1 File Supporting information document contains Table A and Figures A-D. (DOCX)
