# Supplementary material for: Plant water potential improves prediction of empirical stomatal models
Source: PLoS One. 2017 Oct 12;12(10):e0185481. doi: 10.1371/journal.pone.0185481 (PMC5638234; doi:10.1371/journal.pone.0185481)
Supplement: S1 File — (DOCX) [file pone.0185481.s001.docx]

**Plant water potential improves prediction of empirical stomatal models**

**S1 File: Supplemental Tables and Table Legends**

Table A: Species included in the analysis with their biome (needleleaf evergreen temperate (NET), broadleaf evergreen temperate (BET), broadleaf deciduous temperate (BDT), tropical deciduous (TPD), and tropical evergreen (TPE), sample size (N), and the reference study.

| Species name | N | Biome | Reference |
| --- | --- | --- | --- |
| *Acer campestre* | 41 | BDT | (Li *et al.*, 2016) |
| *Acer pseudoplatanus* | 39 | BDT | (Li *et al.*, 2016) |
| *Alphitonia excelsa* | 173 | TPE | (Choat *et al.*, 2006) |
| *Anacardium excelsum* | 14 | TPD | (Meinzer *et al.*, 2004) |
| *Annona hayesii* | 46 | TPD | (Wolfe *et al.*, 2016) |
| *Astronium graveolens* | 338 | TPE | (Wolfe *et al.*, 2016) |
| *Austromyrtus bidwillii* | 35 | TPE | (Choat *et al.*, 2006) |
| *Brachychiton australis* | 100 | TPD | (Choat *et al.*, 2006) |
| *Bursera simaruba* | 104 | TPD | (Wolfe *et al.*, 2016) |
| *Carpinus betulus* | 48 | BDT | (Li *et al.*, 2016) |
| *Cavanillesia platanifolia* | 41 | TPD | (Wolfe *et al.*, 2016) |
| *Cochlospermum gillivraei* | 75 | TPD | (Choat *et al.*, 2006) |
| *Cojoba rufescens* | 319 | TPE | (Wolfe *et al.*, 2016) |
| *Cordia alliodora* | 18 | TPD | (Meinzer *et al.*, 2004) |
| *Corylus avellana* | 35 | BDT | (Li *et al.*, 2016) |
| *Eucalyptus globulus* | 73 | BET | Hernandez et al. 2016 |
| *Ficus insipida* | 14 | TPE | (Meinzer *et al.*, 2004) |
| *Fraxinus excelsior* | 40 | BDT | (Li *et al.*, 2016) |
| *Genipa americana* | 109 | TPD | (Wolfe *et al.*, 2016) |
| *Juniperus monosperma* | 576 | NET | (LIMOUSIN *et al.*, 2013) |
| *Juniperus osteosperma* | 34 | NET | (Koepke & Kolb, 2013) |
| *Phillyrea angustifolia* | 17 | BET | (Resco *et al.*, 2009) |
| *Picea abies* | 544 | NET | (Chmura *et al.*, 2016) |
| *Pinus edulis* | 511 | NET | (LIMOUSIN *et al.*, 2013) |
| *Pinus ponderosa* | 146 | NET | (Koepke & Kolb, 2013) |
| *Pistacia lentiscus* | 23 | BET | (Resco *et al.*, 2009) |
| *Populus balsamifora* | 29 | BDT | (Arango-Velez *et al.*, 2011) |
| *Populus tremuloides* | 43 | BDT | (Anderegg, 2012) |
| *Prosopis velutina* | 23 | BDT | (Lin *et al.*, 2015) |
| *Quercus douglasii* | 166 | BET | (Xu & Baldocchi, 2003) |
| *Quercus gambelii* | 12 | BDT | (Koepke & Kolb, 2013) |
| *Quercus ilex* | 110 | BET | (Martin-StPaul *et al.*, 2012) |
| *Schefflera morototoni* | 19 | TPE | (Meinzer *et al.*, 2004) |
| *Tapirira guianensis* | 33 | TPE | (Meinzer *et al.*, 2004) |

**Supplemental Figures and Figure Legends**

Figure A: Partial dependencies of stomatal conductance (note that Y-axes are unitless because they are model-dependent) on photosynthesis (Photo; umol*m^-2^*s^-1^), vapor pressure deficit (VPD; kPa), CO_2_ concentration (CO2S; ppm) and leaf water potential (LWP; MPa) from the RandomForest model for *Juniperus monosperma* as an example species. Lower right indicates the predicted versus observed plot of stomatal conductance when comparing to out-of-bag predictions – R^2^ of 0.876.

Figure B: Partial dependencies of stomatal conductance (note that Y-axes are unitless because they are model-dependent) on photosynthesis (Photo; umol*m^-2^*s^-1^), vapor pressure deficit (VPD; kPa), CO_2_ concentration (CO2S; ppm) and leaf water potential (LWP; MPa) from the RandomForest model for *Astronium graveolens* as an example species (note this species experienced no variation in CO_2_ concentration). Lower right indicates the predicted versus observed plot of stomatal conductance when comparing to out-of-bag predictions – R^2^ of 0.717.

Figure C: Partial dependencies of stomatal conductance (note that Y-axes are unitless because they are model-dependent) on photosynthesis (Photo; umol*m^-2^*s^-1^), vapor pressure deficit (VPD; kPa), CO_2_ concentration (CO2S; ppm) and leaf water potential (LWP; MPa) from the RandomForest model for *Genipa americana* as an example species (note this species experienced no variation in CO_2_ concentration). Lower right indicates the predicted versus observed plot of stomatal conductance when comparing to out-of-bag predictions – R^2^ of 0.697.

Figure D: Partial dependency of stomatal conductance on leaf water potential for three species in which the functional form was uninterpretable. Species are: *Populus tremuloides* (POTR), *Brachychiton australis* (BAU), and *Quercus gambelii* (QUGA).

**References**

Anderegg W (2012) Complex aspen forest carbon and root dynamics during drought. *Climatic Change*, **111**, 983–991.

Arango-Velez A, Zwiazek JJ, Thomas BR, Tyree MT (2011) Stomatal factors and vulnerability of stem xylem to cavitation in poplars. *Physiologia Plantarum*, **143**, 154–165.

Chmura DJ, Guzicka M, McCulloh KA, Żytkowiak R (2016) Limited variation found among Norway spruce half-sib families in physiological response to drought and resistance to embolism. *Tree physiology*, tpv141.

Choat B, Ball MC, Luly JG, Donnelly CF, Holtum JA (2006) Seasonal patterns of leaf gas exchange and water relations in dry rain forest trees of contrasting leaf phenology. *Tree Physiology*, **26**, 657–664.

Koepke DF, Kolb TE (2013) Species variation in water relations and xylem vulnerability to cavitation at a forest-woodland ecotone. *Forest Science*, **59**, 524–535.

Li S, Feifel M, Karimi Z, Schuldt B, Choat B, Jansen S (2016) Leaf gas exchange performance and the lethal water potential of five European species during drought. *Tree physiology*, **36**, 179–192.

LIMOUSIN J, Bickford CP, Dickman LT et al. (2013) Regulation and acclimation of leaf gas exchange in a piñon–juniper woodland exposed to three different precipitation regimes. *Plant, Cell & Environment*, **36**, 1812–1825.

Lin Y-S, Medlyn BE, Duursma RA et al. (2015) Optimal stomatal behaviour around the world. *Nature Climate Change*, **5**, 459–464.

Martin-StPaul NK, Limousin J-M, Rodríguez-Calcerrada J, Ruffault J, Rambal S, Letts MG, Misson L (2012) Photosynthetic sensitivity to drought varies among populations of Quercus ilex along a rainfall gradient. *Functional Plant Biology*, **39**, 25–37.

Meinzer FC, James SA, Goldstein G (2004) Dynamics of transpiration, sap flow and use of stored water in tropical forest canopy trees. *Tree Physiology*, **24**, 901–909.

Resco V, Ewers BE, Sun W, Huxman TE, Weltzin JF, Williams DG (2009) Drought-induced hydraulic limitations constrain leaf gas exchange recovery after precipitation pulses in the C3 woody legume, Prosopis velutina. *New Phytologist*, **181**, 672–682.

Wolfe BT, Sperry JS, Kursar TA (2016) Does leaf shedding protect stems from cavitation during seasonal droughts? A test of the hydraulic fuse hypothesis. *New Phytologist*, **212**, 1007–1018.

Xu L, Baldocchi DD (2003) Seasonal trends in photosynthetic parameters and stomatal conductance of blue oak (Quercus douglasii) under prolonged summer drought and high temperature. *Tree physiology*, **23**, 865–877.
